# The importance of patient compliance in repeated rounds of mass drug administration (MDA) for the elimination of intestinal helminth transmission

**DOI:** 10.1186/s13071-017-2206-5

**Published:** 2017-06-12

**Authors:** Sam H. Farrell, James E. Truscott, Roy M. Anderson

**Affiliations:** 1 London Centre for Neglected Tropical Disease Research, Department of Infectious Disease Epidemiology, St Mary’s Campus, Imperial College London, London, W2 1PG UK; 2 The DeWorm3 Project, The Natural History Museum of London, London, SW7 5BD UK

**Keywords:** Soil-transmitted Helminths, Schistosomiasis, Mass Drug Administration, Compliance, Systematic non-compliance, Mathematical modelling

## Abstract

**Background:**

Systematic non-compliance to chemotherapeutic treatment among a portion of the eligible population is thought to be a major obstacle to the elimination of helminth infections by mass drug administration (MDA). MDA for helminths is repeated at defined intervals such as yearly or every 2 years, as a consequence of the inability of the human host to develop fully protective immunity to reinfection. As such, how an individual complies to these repeated rounds of MDA can have a significant impact on parasite transmission. The importance of this factor is poorly understood at present. Few epidemiological studies have examined longitudinal trends in compliance in the many communities in areas of endemic helminth infection that are undergoing MDA. Reducing systematic non-compliance will obviously increase the number of individuals treated, but it may also alter the dynamics of parasite transmission.

**Methods:**

Here we develop an individual-based stochastic model of helminth transmission and MDA treatment to investigate how different patterns of compliance influence the impact of MDA for two groups of helminths, the soil transmitted nematode infections and the schistosome parasites. We study the effect of several alternative treatment and compliance patterns on the dynamics of transmission.

**Results:**

We find that the impact of different compliance patterns, ranging from random treatment at each round of chemotherapy to systematic non-compliance by a proportion of the population, is very dependent on both transmission intensity in a defined setting and the type of infection that the treatment is targeted at. Systematic non-compliance has a greater impact on the potential for elimination of *Schistosoma mansoni* transmission by intensive MDA, than it does on *Ascaris lumbricoides*.

**Conclusions:**

We discuss the implications of our findings for the prioritisation of resources in MDA programmes and for monitoring and evaluation programme design. The key message generated by the analyses is that great care must be taken to record individual longitudinal patterns of compliance at each round of MDA as opposed to just recording overall coverage.

**Electronic supplementary material:**

The online version of this article (doi:10.1186/s13071-017-2206-5) contains supplementary material, which is available to authorized users.

## Background

Over the past decade the neglected tropical diseases (NTDs) have become a major focus for research, both on new treatments and diagnostics and on the best method to reduce or eliminate parasite transmission by various interventions [1, 2]. Of these, parasitic infections by soil-transmitted helminths (STH) and blood flukes of the genus *Schistosoma*, are among the most common, with estimates of those harbouring STH infections ranging up to 2 billion individuals worldwide [3, 4].

STH infections are caused by a variety of intestinal nematode species. The most important are the roundworm *Ascaris lumbricoides*, the hookworms *Ancylostoma duodenale* and *Necator americanus* and the whipworm *Trichuris trichiura*. We will focus here on *A. lumbricoides*, the most common of these in humans [5]. Mild infections are often symptomless but heavier infections may lead to serious morbidity including diarrhoea, and may cause or exacerbate nutritional deficiencies leading to reduced growth in children [6]. The disease schistosomiasis, caused by infection with the schistosome parasites, is widespread primarily across sub-Saharan Africa, occurring also in the Americas, the Eastern Mediterranean region, Southeast Asia and the Western Pacific, affecting primarily rural communities in contact with contaminated water sources. Heavy infections with this parasite can cause serious morbidity and even mortality [7]. Though both *Schistosoma mansoni* and *Schistosoma haematobium* are widespread in humans, we focus on intestinal schistosomiasis through *S. mansoni* infection in comparison with the intestinal helminth *A. lumbricoides*.

Current WHO guidelines aim to eliminate STH as a public health problem in children by 2020 through a focus on preschool-aged children (pre-SAC, 2–4 year olds) and school-aged children (SAC, 5–14 year olds) though mass drug administration (MDA) of albendazole or mebendazole at a coverage of at least 75% of both preschool and school-aged children [6] . Similarly, the WHO goals for schistosomiasis are to control morbidity through an MDA treatment regime in affected areas covering at least 75% of school-aged children by 2020, as well as at risk adults, with an eventual goal of elimination as a public health problem by 2025 [7].

If MDA is aimed at elimination the transmission of helminth infection in defined settings, sufficient treatment coverage and frequent treatment are essential as outlined in a series of recent publications [8–13]. There are a number of potential social, logistical and technical challenges in achieving high coverage [14, 15]. A large gap can exist between the proportion of the population actually participating in MDA (having actually taken the relevant drug), and the coverage reported by government or international agencies [16]. Reasons for non-compliance (for the definitions of compliance and coverage as used here, see below) can vary very widely even within the same treatment programme, for the same infection, within the same country [17]. These might include access issues, programme fatigue for longer-running treatment programmes, whether the drug distributers are personally known to the local population, and education of the population to causes of disease and the benefits of MDA [16–18]. MDA programmes that are designed to account for those social and behavioural factors that act to reduce compliance to treatment, can greatly improve treatment coverage [18].

Treatment coverage and compliance figures are commonly aggregated on a village-wide level or other administrative units, and may hide substantial variation in the numbers of treatments taken by individuals during an MDA programme [16]. Very few studies have included individual treatment data with a longitudinal component to cover what happens at each round of drug administration. There have been increasing efforts to ascertain a picture of compliance with STH treatment that reflects individual compliance over time in addition to simple measures of overall coverage, with two major studies in progress or upcoming that will include longitudinal measurement of compliance [19, 20].

Individuals who systematically do not adhere to treatment, over a number of treatment rounds, which we term *systematic non-compliance*, may provide an important reservoir to sustain reinfection in the population. The effect of systematic non-compliance on transmission has not so far been directly investigated for the major helminth infections of humans [16]. Systematic non-compliance is clearly an issue in terms of preventing individual morbidity. Moreover, any non-compliance is a programmatic issue which determines the population level impact of an MDA intervention.

The importance of the more subtle effects of compliance on the transmission dynamics of helminth parasites is less clear. For example, in the absence of variation in coverage, what effect does systematic non-compliance have on the difficulty of transmission elimination by MDA? An answer to this question would help guide the allocation of resources within international parasite elimination attempts. In a defined transmission intensity setting is it necessary to maximise coverage by any means available, or is the best approach to focus most attention on reaching regularly untreated members of a community in an effort to reduce any reservoir-of-infection effect?

Here, we present a quantitative framework for assessing of the importance of compliance patterns in eliminating transmission. Rather than “elimination as a public health problem” we consider scenarios in which complete elimination of the parasite from a small community is the goal and analyse the likelihood of success.

We undertake a computational analysis of the impact of compliance pattern and systematic non-compliance in communities with defined intrinsic transmission intensities and treatment patterns, in which either *Ascaris lumbricoides* or *Schistosoma mansoni* parasites are endemic. By focusing on a pair of disease and treatment settings we provide a snapshot of the variability of compliance impact as well as considering the predicted impact on disease elimination in these particular settings.

In published mathematical models of transmission dynamics and MDA a common assumption is that treatment is administered at random to the population at each round, but with a defined proportion of the population receiving treatment at each round [21]. Plaisier et al., in a paper based on an individual based simulation model [22], argue that attendance patterns under randomly-allocated treatment do not adequately reflect actual attendance in real world situations, as individuals may well participate in treatment irregularly. This study does not directly compare simulation results under alternative attendance patterns. To address this gap we make comparisons between a proposed alternative model of attendance pattern and random attendance in both diseases, using a well-defined individual based stochastic model which incorporates the known population biology of the parasites. Efforts to assess and improve models of realistic treatment patterns are ongoing [23].

## Methods

### Models of helminth transmission

An individual based stochastic model framework allows for heterogeneity at the individual level and tracking of individual behaviour which influence both exposure to infection and compliance to treatment. The model builds on the framework described in an earlier publication by Anderson & Medley [24]. Much recent work on the impact of MDA on helminth infections has focussed on the predictions of a hybrid deterministic partial differential equation-based model, describing changes in mean worm burden age profile in the human population over time [11, 24], which permits the calculation of treatment coverage levels that will eliminate parasite transmission. These models include probabilistic elements (in terms of a distribution of parasite numbers per host of negative binomial form, with fixed aggregation parameter *k*), density dependence in fecundity and sexual mating for the dioecious parasites (assumption of polygamy for STH and monogamy for schistosomes). As noted earlier, these models assume that for a defined level of coverage, treatment is at random at each round, and with a constant probability.

The deterministic and stochastic models share a number of common features. In brief, due to generally strong age dependence in observed helminth infection intensities both models account for age-specific contact rates. Specifically, the transmission parameter $\upbeta$ represents factors including time spent in contact with infectious material or degree of personal protection from infection that vary with age (e.g. use of sandals).

Of particular importance is the presence of density-dependent egg production controlled by the parameter $\upgamma .$Increasing numbers of worms lead to a reduction in per-female egg output, which is the key limiting factor to overall parasite population.

A second density-dependent effect, the worms’ sexual reproduction, leads to a predicted breakpoint in transmission dynamics [25, 26]. Where numbers of worms are low, the likelihood of both sexes being present in a host drops, leading to either loss of egg production or the production of unfertilised eggs, depending on species. This means that below a certain context-dependent threshold, the parasite population cannot sustain itself and collapses toward extinction without further intervention. This effect is highly dependent on the degree of worm aggregation across hosts and is of particular importance in the context of regular MDA and parasite elimination [21, 24].

Reproduction may be either monogamous or polygamous. The soil-transmitted helminths are thought to be polygamous and the schistosomes are thought to pair for life, and hence are monogamous. Hard evidence in either case is very limited. Fertile eggs produced contribute to a single environmental reservoir of infection, symbolising infectious material across a village, single water source or similar local area. In the absence of directly observed data on age-specific contribution to the infectious reservoir, we assume contribution rates $\uprho$ are equal to age-specific contact rates.

Treatment is by periodic MDA, and has an immediate impact in reducing worm burden. Evidence for possible host immune responses to infection as a consequence of past exposure is limited at present and is not modelled explicitly. Many immunological responses to infection can be observed in terms of antibody and cellular responses to parasite antigens but these do not create and effective acquired immunity. However, in the stochastic model genetic/behavioural differences between individual hosts are included in host predisposition to infection, as described below.

### Individual-based stochastic model

The stochastic model used throughout the analyses presented in this paper incorporates a number of additional features over those described above. Hosts are modelled individually with their own burden of male and female worms. The acquisition and deaths of individual worms in individual hosts are modelled as distinct events, as are births and deaths within the host population.

**Fig. 1:**
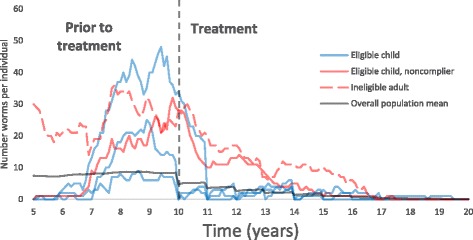
An illustrative selection of worm burdens over time in five individuals set against the mean worm burden in the human population. *X-axis*: year of simulation. *Y-axis*: the number of parasites per individual host. Part of the population is treated and benefits immediately while untreated individuals benefit over time via ‘herd effects’ through natural worm mortality as infectious material in the environment is reduced. Four children are born in year five and are eligible for treatment. Three are treated under a semi-systematic treatment setting, and attend most treatments. One systematically does not comply with treatment. One adult is ineligible and does not receive treatment. Treatments are annual with eight rounds from year 10 onwards (parameter values as defined in Table 2)

The basic behaviour and basic outputs of the two models are roughly equivalent; quantification of the dynamics over time of helminth transmission and predictions on elimination, impact of control measures and so on. The benefit of the individual-based stochastic approach is that a number of additional features may be implemented which are difficult or impossible to produce otherwise. Different compliance settings for instance may be modelled using a deterministic model but require additional compartments, an approach that quickly becomes unmanageably complex. A stochastic approach allows us to quantify the degree of variability in predicted outcomes, in particular the probability of disease elimination rather than a binary success/failure prediction when using a deterministic framework. In addition this individual-based approach allows us to view details that are impossible to visualise employing a deterministic approach. An example of the time trajectories of parasite burden in 5 individual people within a stochastic simulation is shown as an illustrative example in Fig. 1. Note how individual trajectories vary widely from the mean behaviour.

Worm burden data shows that worms are not distributed evenly or randomly (Poisson) through the host population and instead tend to be aggregated more highly in some individuals than others [27, 28]. To account for differential exposure to infection due to a range of possible host genetic, immunological, behavioural, social or environmental factors, individuals are assigned a personal predisposition index to infection, drawn from at random from a gamma distribution at birth with shape parameter $\upalpha$ [21, 25]. Given worm death rates that are constant over time, and the Poisson distribution of infection events, the distribution of worms that arises across the host population is a negative binomial in form due to the compounding of Poisson distributions in individuals with means distributed in a gamma form. This overall distribution of parasite numbers per host matches observed parasite aggregation data well [28]. Note however, that the aggregation parameter, *k*, of the negative binomial will vary over time as the mean burden and the prevalence change due to chance events in the acquisition and loss of parasites and births and deaths of the human host. In other words, the value of *k* is dynamic. It also varies across the age classes due to their differing age dependent infection rates as described above.

Egg produced contribute to an environmental reservoir of infection. Individuals contribute to the pool of infection according to their female worm burden, subject to the presence of males, at a rate in accordance with their age-specific contact rate. Because parameters describing the detailed reproductive steps (e.g. for reproduction via snail vectors in the case of schistosomiasis) are of poor quality or missing entirely we retain a straightforward deterministic model for the environmental reservoir.

**Fig. 2:**
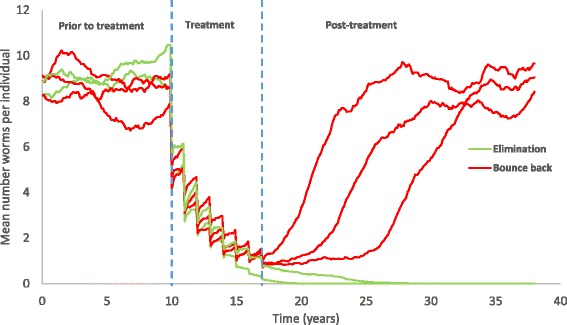
An illustration of five simulation repeats, showing stochastic variability and the impact of treatment. X-axis: year of simulation. Y-axis: the mean number of parasites in each independent population. In two repeats, elimination is successful, in three others the parasite population bounces back

As before, a breakpoint due to sexual reproduction is present in the transmission dynamics with two stable states, endemic infection or parasite extinction, separated by an unstable equilibrium. In this case, the endemic state is subject to considerable stochastic variation over time. The same variability, when close to the breakpoint, introduces an additional uncertainty into the dynamics in this region (Fig. 2).

To take account of such effects, we run a large number of repeat simulations (one thousand repeats) using identical parameters. Most of the results presented here are derived from the mean across repeats, allowing quantification of the overall behaviour while still accounting for stochastic (= chance) effects producing a range of outcomes. Probabilities of elimination under the specified conditions are equal to the proportion of repeats which result in extinction of both parasites and infectious material in the long term within the defined habitat.

While treatment in the deterministic model necessarily combines drug efficacy and treatment coverage, an individual-based model can separate these factors. Drug efficacy is given a single value describing the probability of worm mortality as a result of treatment.

The stochastic simulations are event-driven. At any given time point, the time step to the next event is randomly drawn from an exponential distribution with the parameter of the exponent given by the rate at which an event occurs. These rates are defined in Table 1.

**Table 1:** Model events, adapted from [11], where *N*_*i*_ is a host’s total worm burden, of which *n*_*i*_ are female worms, *Ber()* is a Bernoulli-distributed random variable and *δ()* is the Dirac delta function

Event	Definition	Rate
Worm acquisition by host *i*, aged *a*	${\mathrm{N}}_{\mathrm{i}}\to {\mathrm{N}}_{\mathrm{i}}+1$ ${\mathrm{n}}_{\mathrm{i}}\to {\mathrm{n}}_{\mathrm{i}}+\mathrm{B}\mathrm{e}\mathrm{r}(0.5)$	$\upbeta \left({\mathrm{a}}_i\right){\uplambda}_{\mathrm{i}}\mathrm{L}$ * per host per unit of time*
Worm death in host *i*	${\mathrm{N}}_{\mathrm{i}}\to {\mathrm{N}}_{\mathrm{i}}-1$ ${\mathrm{n}}_{\mathrm{i}}\to {\mathrm{n}}_{\mathrm{i}}-\mathrm{B}\mathrm{e}\mathrm{r}(0.5)$	$\upsigma$ * per worm per unit of time*
Host birth/death for host aged *a*	At death, host is replaced with a newborn; ${\mathrm{N}}_{\mathrm{i}}={\mathrm{n}}_{\mathrm{i}}=0$ and all host variables reset.	$\upmu \left({\mathrm{a}}_{\mathrm{i}}\right)$ * per unit of time*
Treatment of host *i*, aged *a*	${\mathrm{N}}_{\mathrm{i}}={\mathrm{N}}_{\mathrm{i}}*\left(1-\left[\mathrm{drug}\;\mathrm{eff}.\right]\right)$	$\updelta \left(\mathrm{t}-{\mathrm{t}}_{\mathrm{j}}\right)\mathrm{g}\left({\mathrm{a}}_{\mathrm{i}}\right)$

The population biology parameters employed in the simulations described in the results section are as defined in Table 2 for both *Ascaris lumbricoides* and *Schistosoma mansoni.* These derive from field epidemiological studies in India (*A. lumbricoides*) and Kenya (*S. mansoni*). The methods used to obtain these estimates are described in [11] and [10].

**Table 2:** Parameter definitions and values. $\beta$ and *ρ* are stated across the age groups 0–2, 2–5, 5–15, 15–70+. Parameter values as [11] for *Ascaris* and [10] for *S. mansoni*

Parameter (units)	Definition	Value: *A. lumbricoides*	Value: *S. mansoni*
${\mathrm{R}}_0$	Basic reproductive number or ratio	2.12	1.55
$\upbeta, \uprho$	Relative contact rates, relative contribution to the environmental reservoir	0.22, 1.88, 1, 0.53	0.036, 0.17, 1, 0.069
$\upgamma$	Egg production density dependence parameter	0.07	0.0006
$\mathrm{k}$	Shape parameter of negative binomial distribution of worms amongst hosts	0.9	0.24
$\upsigma$ (/year)	Inverse of worm lifespan	1	0.1754
$\uplambda$ (epg/female worm)	Net egg output per female worm	3983	0.14
reservoir decay rate (/year)	Rate of decay of eggs in the environmental reservoir	6	3
drug efficacy	Proportion of a host’s worms removed by a single treatment	99% (Albendazole)	86% (Praziquantel)

### Treatment and compliance

The model permits tailoring of individual treatment over multiple rounds of administration. Here treatment refers to treated age groups as well as a compliance pattern. We consider three types of compliance patterns: *random compliance*, in which the attending individuals are selected randomly at each treatment round; *systematic non-compliance*, in which individuals are assigned a lifelong “complier” or “non-complier” status and thus either attend all treatments or none; and *semi-systematic* compliance as an intermediary between the two previous patterns.

Under a semi-systematic compliance setting individuals each attend treatment according to a lifetime propensity to comply with treatment - capturing the effect of a range of personal and sociological factors, such as family circumstance or physical difficulty of access to treatment. We follow a formulation by Plaisier and colleagues [22] (see also Additional file 1: Figure S1). Each individual is randomly assigned a lifelong attendance factor *a*_*i*_ from a uniform distribution on the interval 0–1. At each treatment, the individual’s probability of attending is *a*_*i*_^*(1-C)/C*^, where *C* is the overall coverage. A property of this formulation is that at each treatment the average likelihood of attending across the eligible population is equal to *C* and so overall coverage is consistent between treatment scenarios.

We assume that both of the parasites investigated will be treated according to disease-specific WHO guidelines. Coverage to account for schistosomiasis treatment of “at risk” adults is not specified by WHO and is here set at 30%, following deterministic predictions of what level of adult coverage may result in interrupting parasite transmission for the defined intrinsic level of transmission in the community (the value of R_0_ in Table 2). Rather than covering every possible situation we thereby produce several alternative scenarios in order to investigate the variety of impacts that compliance and systematic non-compliance may have.

### A note on terminology

The use and meaning of the terms *compliance*, *coverage* and *elimination* varies in the published epidemiological literature [16], and these terms may be used differently in different country treatment guidelines. Unless stated otherwise, definitions applied here are as follows;

***Coverage***: the proportion of the whole eligible population that actually receives treatment at a given treatment round. This definition is sometimes referred to elsewhere as *compliance* to distinguish between treatment being allocated and actually being taken. It is of fundamental importance that “true” coverage is measured accurately in treatment programmes. However, there is no need for a distinction in our computational model of transmission.

***Systematic non-compliance***: a proportion of the population remains untreated across consecutive treatment rounds. For modelling purposes, we do not distinguish between the reasons treatment was not taken by any individual.

***Elimination***: the WHO’s guidelines on STH and schistosomiasis make reference to *elimination as a public health problem*. This requires only highly effective control and is not the same as disease elimination in the formal sense of having reduced transmission of disease to zero in a specific area [29]. Though we use the WHO’s guidelines on treatment in our modelling, *elimination* in our context is in the more rigorous sense, breaking transmission without reoccurrence of infection in simulated settings.

## Results

### Impact of systematic non-compliance

The simulations well illustrate the view that individuals who systematically do not comply with MDA treatment over a number of years, may provide a reservoir of infection in the population. However, while systematic non-compliance may be an issue in individual morbidity where infections go untreated, and equally clearly non-compliance is a programmatic issue insofar as achievable coverage is reduced, it does not necessarily follow that systematic non-compliance will substantially interfere with elimination efforts through its impact on transmission dynamics alone if coverage is well above the level required to break transmission as predicted by deterministic models.

Focusing on the WHO targets for treatment of 75% coverage for eligible children, we examine a worst case scenario in which one portion of the population is treated at each round, and another comprises systematically non-complying individuals who never receive treatment.

**Fig. 3:**
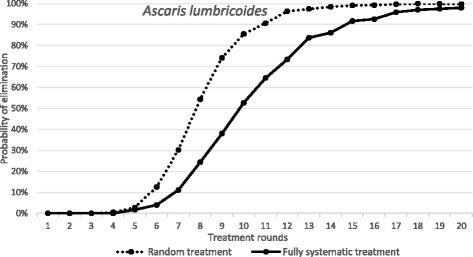
The dependence of probability of elimination of *A. lumbricoides* on the number of annual treatment rounds. *X-axis*: the number of treatment rounds. *Y-axis*: the proportion of 1000 repeats in which elimination is achieved, in a random treatment scenario (all individuals treated at random, 75% pre-SAC and SAC coverage) and a fully systematic treatment scenario (75% of pre-SAC and SAC always treated, remaining population never treated). Parameter values as defined in Table 2

**Fig. 4:**
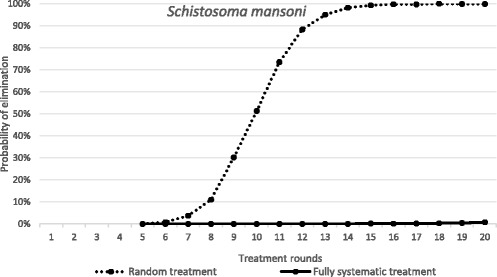
The dependence of probability of elimination of *S. mansoni* on the number of annual treatment rounds. *X-axis*: the number of treatment rounds. *Y-axis*: the proportion of 1000 repeats in which elimination is achieved, in a random treatment scenario (all individuals treated at random, 75% SAC and 30% adult coverage) and a fully systematic treatment scenario (75% SAC and 30% adults always treated, remaining population never treated). Parameter values as defined in Table 2

The results are presented in Fig. 3 for *Ascaris* and in Fig. 4 for *S. mansoni* as the probability of the interruption of transmission and parasite elimination after various rounds of treatment at a fixed coverage levels for random and systematic non-compliance. The different parasites exhibit different responses when the non-compliance scenario is compared with random treatment, In the case of *Ascaris* the negative impact is notable but perhaps modest. The simulations based on the stochastic models suggest that breaking transmission is still possible in a reasonable time-frame, requiring only a modest increase in the number of rounds (Fig. 3). For example, after 10 rounds of treatment the probability of elimination is roughly 90% in the case of random treatment at each round, while for systematic non-compliance it is just of 50% for the identical number of rounds of treatment. Given that this an extreme non-compliance setting - 25% of the population never attends - it would seem that the impact on transmission dynamics of any reservoir-of-infection effect on the chances of elimination within a local area given a well-mixed population (any one person spreads infective stages throughout the population’s habitat) is relatively limited. In part this is related to the magnitude of R_0_ (it is a moderate transmission intensity with a value of 2.12), and in part the short lifespan of the parasite in humans (1 year) reduces the impact of non-compliers providing a reservoir of infection.

In contrast as shown in Fig. 4, in an *S. mansoni*-endemic area, the impact of the same systematic compliance pattern is far greater, with elimination essentially not achievable even after 20 rounds of annual treatment. Note that this results even when the magnitude of R_0_ places this example in a low transmission setting with R_0_ = 1.55 (Table 2).

Although a number of factors determine the differences in transmission dynamics between species, parasite lifespan is a very strong influence in the differential effect of systematic non-compliance. For example, it has a major influence on the ‘bounce back’ time post cessation of MDA, with longer lifespan parasites bouncing back more slowly [21]. In the context of compliance to treatment long parasite lifespan would allow for a longer-lasting reservoir of infection in systematically non-complying sections of the population. It is difficult to isolate the effect of parasite lifespan within models of parasite transmission dynamics because of its central role in determining the magnitude of the basic reproductive number, R_0_. Reproductively mature adult worm lifespan lies in the numerator (the death rate is in the denominator) of R_0_, and hence without adjusting other parameter values, such as the infection rate, β.

The results presented in Fig. 4 for *S. mansoni* serve to illustrate how important individual compliance over sequential rounds of treatment is to determining the impact of MDA, as opposed to simple measures of coverage as commonly recorded by government health departments in endemic regions.

### Impact of partial non-compliance to treatment

**Fig. 5:**
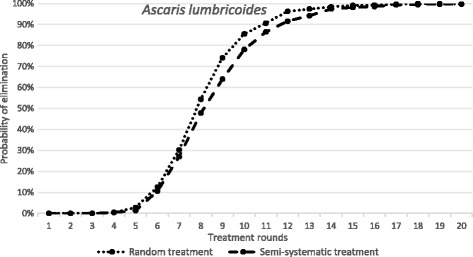
The dependence of probability of elimination of *A. lumbricoides* on the number of annual treatment rounds. *X-axis*: the number of treatment rounds. *Y-axis*: the proportion of 1000 repeats in which elimination is achieved, in a random treatment scenario (all individuals treated at random, 75% pre-SAC and SAC coverage) and a semi-systematic treatment scenario (individuals attend according to a personal propensity, 75% pre-SAC and SAC coverage). Parameter values as defined in Table 2

There have been a few published studies that attempt to ascertain a picture of patterns of individual compliance to MDA in a longitudinal context [16]. As outlined in the methods section Plaisier and colleagues [22] discuss a formulation for a compliance pattern - termed semi-systematic compliance - which takes into account the personal propensity of individual members of a population to attend successive MDA treatment rounds [22]. We compare this formulation to an assumption of randomly-allocated treatment with no personal propensity to non-compliance. As illustrated in Figs. 5 (*Ascaris*) and 6 (*S. mansoni*) by reference to the probability of elimination post a given number of rounds of treatment at the WHO target level of 75% coverage for Pre-SAC and SAC, the size of the effect of compliance pattern on the probability of elimination depends strongly on the type of infection being targeted by MDA. While the duration of treatment required to eliminate *Ascaris* is nearly identical under both compliance patterns, elimination of *S. mansoni* requires a considerably longer treatment programme under a semi-systematic compliance pattern. However, note that elimination of *Ascaris* is possible for a partial non-compliance pattern after many rounds of treatment.

**Fig. 6:**
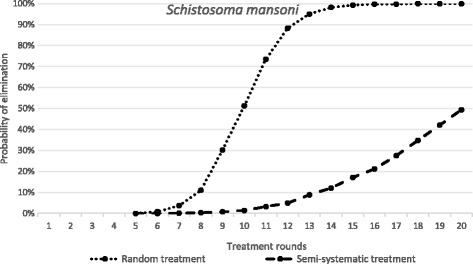
The dependence of probability of elimination of *S. mansoni* on the number of annual treatment rounds. *X-axis*: the number of treatment rounds. *Y-axis*: the proportion of 1000 repeats in which elimination is achieved, in a random treatment scenario (all individuals treated at random, 75% SAC and 30% adult coverage) and a semi-systematic treatment scenario (individuals attend according to a personal propensity, 75% SAC and 30% adult coverage). Parameter values as defined in Table 2

### Indirect benefits of MDA programmes for non-compliers

**Fig. 7:**
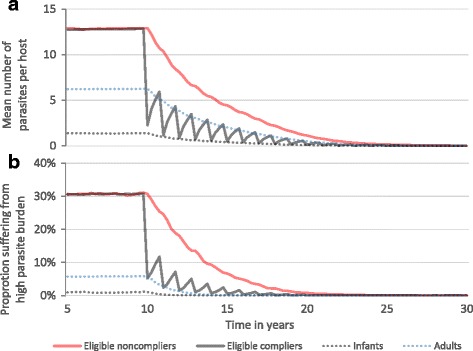
Grouped parasite burdens in an *A. lumbricoides*-endemic area. Pre-SAC and SAC are eligible for annual treatment at 75% coverage. For illustration, treatment continues indefinitely. *X-axis*: year of simulation. *Y-axis*: across 1000 repeat simulations; (**a**) mean parasite burden, (**b**) proportion of each group suffering from high worm burden. Parameter values as defined in Table 2. The definition of high parasite burden is given in [32]

**Fig. 8:**
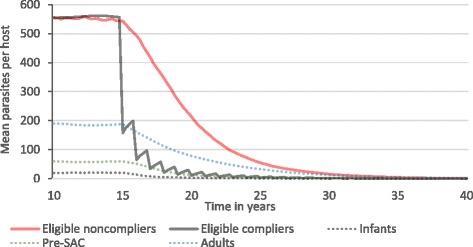
Grouped mean parasite burdens in an *S. mansoni*-endemic area. SAC are eligible for annual treatment at 75% coverage. For illustration, treatment continues indefinitely. *X-axis*: year of simulation. *Y-axis*: mean parasite burden across 1000 repeat simulations. Parameter values as defined in Table 2

Systematically non-complying members of the population will still benefit from decreases in population-wide infection burden (in a population exposed to a single pool of infectious material), due to the reduction in the pool of infectious material due to treatment in the compliers. Reductions in egg production in the treated population are sufficient to reduce rates of infection in all individuals (Fig. 7) over time. These ‘indirect benefits’ are realised at a pace that is surprisingly rapid as illustrated in Fig. 7 for *Ascaris* and Fig. 8 for *S. mansoni*. These figures record both the mean worm burden per host and the proportion of untreated (pre-SAC and SAC) children suffering from a high parasite burden at the start of MDA. This latter variable roughly declines by 50% over the first 18 months of treatment and 50% again over the following 18 months (Figs. 7 and 8).

## Discussion

Systematic non-compliance to MDA treatment to control helminth infections may be important in some circumstances and for specific types of infection. Clearly, non-compliance is an important factor to be addressed in efforts to increase treatment coverage especially if in the coming years health policy targets shift from morbidity control to transmission elimination. However, the impact of attendance patterns, rather than simple coverage, is poorly understood in many health policy circles. A key question is should the priority be placed on reaching all members of a community, or simply on maximising coverage regardless of who is treated? The simulation results reported in this paper begin to provide a template for answering this important question.

Our findings indicate that the answer is conditional on the helminth infection being targeted and on the underlying transmission intensity in a defined location. MDA aimed at *A. lumbricoides* elimination would likely benefit much less from the direct targeting of systematically non-complying individuals than is the case for treatment programmes for *S. mansoni* infection. In the former case the same resources would be better focussed on increasing coverage by whatever means available. Conversely it seems clear that reaching as many untreated individuals as possible should be made a particularly high policy priority when the aim is *S. mansoni* elimination. In both the parasites examined in our simulation scenarios, adult parasite lifespan is a key consideration. For long lifespans, non-compliers output infective stages into the environment for long periods. This is the case for schistosomes and importantly for the filarial worms which may have lifespans of around 10 years [21].

Within the soil transmitted nematode species the lifespans of *Ascaris* and *Trichuris* are relatively short, estimated at around 1-2 years and 2-3 years respectively [30], while that of the two hookworm species are estimated variously at around 1–3 years (*Ancylostoma duodenale*), 3–10 years (*Necator americanus*) to as much as 18 years [31]. This would suggest for this group of helminths, systematic non-compliance is of greater importance for hookworm than the other species.

The optimum route to transmission elimination may therefore vary between helminth species, even within the soil-transmitted helminths, depending on the degree of non-compliance and the prevailing transmission intensity in a given location. This will be crucial in deciding whether systematic non-compliance is a problem substantial enough to warrant specific action in elimination efforts for particular helminth infections, as opposed to simply trying to increase overall coverage and compliance. If transmission intensity is high, high coverage will be required over many years to move towards transmission elimination.

In practice, it is generally very difficult to identify individual non-compliers on the ground without detailed studies in monitoring and evaluation programmes that have a longitudinal component following individual behaviour at each round of MDA. In general the major deterrent to detailed longitudinal studies is cost. Even where it is known that an individual has repeatedly missed annual treatment, successive visits by distributors to ensure treatment will often be prohibitively time-consuming and hence costly. A broader rather than targeted strategy of maximising coverage may be more appropriate depending on the parasitic infection and local circumstances.

From an epidemiological research perspective, it is surprising that so few longitudinal studies of compliance of individuals to treatment in MDA programmes, whether for STH, schistosomes, lymphatic filariasis or onchocerciasis, have been undertaken [16]. In order to better model the impact of non-compliance and thus better inform the design of treatment and monitoring and evaluation programmes, a more detailed understanding of who is treated in MDA programmes, and when, is essential. Here we have made necessary assumptions on the treatment pattern, and in the absence of good data examined three simple scenarios; namely, all individuals are either always treated or always untreated, all individuals may be treated with a personal propensity to attend, and all individuals are treated at random. In reality, some proportion of individuals or groups of individuals might remain entirely untreated while the remaining population might be treated in a pattern somewhat akin to a personal attendance propensity. As well illustrated by our analyses, which pattern prevails in a given setting matters, especially for long lived helminth species.

This in turns relates to how we interpret the observed pattern of MDA impact. High coverage may be recorded but if systematic non-compliance prevails in a significant proportion of children, impact on reinfection rates may be limited. Future monitoring and evaluation programmes must pay greater attention to recording not only overall coverage, but also the proportion of people who take treatment at each round it is offered.

## Conclusions

We have described a stochastic individual based model for helminth transmission and MDA treatment. Overall conclusions from this model on elimination time required (rounds of MDA) at various levels of coverage in eligible children are in excellent agreement with earlier findings derived from age structured deterministic models based on sets of partial differential equations [10, 11]. The stochastic models, however, provide considerably greater scope for the inclusion of individual variation between people in exposure to infection and compliance to treatment. They provide a powerful tool for exploring the impact of treatment patterns on how well MDA controls both morbidity and transmission. Future collection of full individual-level compliance data longitudinally over multiple treatment rounds could be used to inform more detailed country- or region-specific models and provide more detailed guidance on optimum treatment.

## Additional file

Additional file 1: Figure S1. Simulated data on “actual” attendances by individuals in simulation throughout an eight-round treatment programme in random, semi-systematic and systematic compliance settings, consistent with distributions derived by Plaisier et al. [22]. (PDF 92 kb)

## Abbreviations

MDA: Mass drug administration
NTDs: Neglected tropical diseases
STH: Soil-transmitted helminths
WHO: World Health Organisation
