# Supplementary figures and images for: The importance of patient compliance in repeated rounds of mass drug administration (MDA) for the elimination of intestinal helminth transmission
Source: Parasit Vectors. 2017 Jun 12;10:291. doi: 10.1186/s13071-017-2206-5 (PMC5469187; doi:10.1186/s13071-017-2206-5)

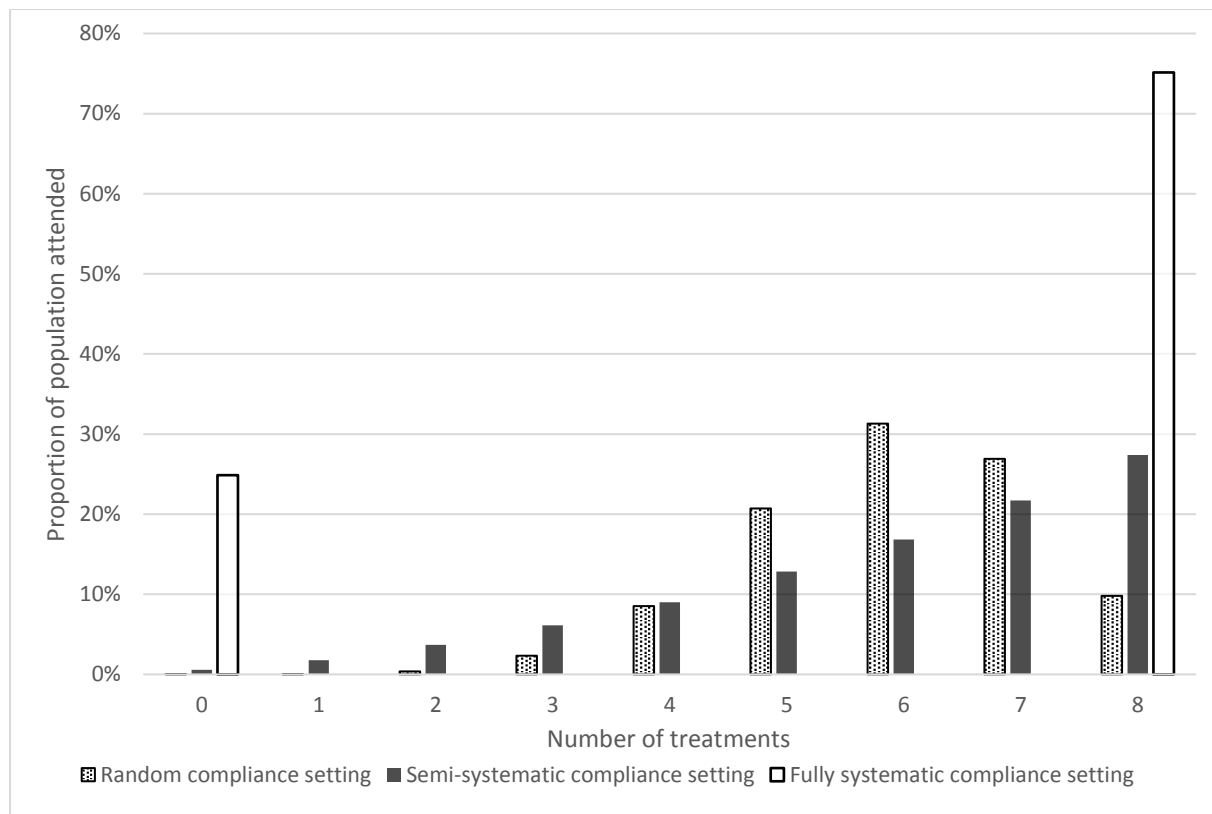

Supplement: Additional file 1: Figure S1. — Simulated data on “actual” attendances by individuals in simulation throughout an eight-round treatment programme in random, semi-systematic and systematic compliance settings, consistent with distributions derived by Plaisier et al. [22]. (PDF 92 kb) [file 13071_2017_2206_MOESM1_ESM.pdf]
